# Supplementary material for: Dynalign II: common secondary structure prediction for RNA homologs with domain insertions
Source: Nucleic Acids Res. 2014 Nov 21;42(22):13939–48. doi: 10.1093/nar/gku1172 (PMC4267632; doi:10.1093/nar/gku1172)
Supplement: SUPPLEMENTARY DATA [file supp_gku1172_nar-02021-z-2014-File012.zip › manual/javadoc/RNAstructure_java_utilities/package-tree.html]

RNAstructure\_java\_utilities Class Hierarchy


JavaScript is disabled on your browser.


- Overview
- Package
- Class
- Tree
- Index
- Help

- Prev
- Next

- Frames
- No Frames

- All Classes

# Hierarchy For Package RNAstructure\_java\_utilities

Package Hierarchies:

- All Packages

## Class Hierarchy

- java.lang.Object
  - RNAstructure\_java\_utilities.BorderBuilder (implements java.io.Serializable)
  - java.awt.Component (implements java.awt.image.ImageObserver, java.awt.MenuContainer, java.io.Serializable)
    - java.awt.Container
      - javax.swing.JComponent (implements java.io.Serializable)
        - javax.swing.AbstractButton (implements java.awt.ItemSelectable, javax.swing.SwingConstants)
          - javax.swing.JMenuItem (implements javax.accessibility.Accessible, javax.swing.MenuElement)
            - javax.swing.JMenu (implements javax.accessibility.Accessible, javax.swing.MenuElement)
              - RNAstructure\_java\_utilities.RNAstructureMenu (implements java.awt.event.ActionListener)
          - javax.swing.JToggleButton (implements javax.accessibility.Accessible)
            - javax.swing.JCheckBox (implements javax.accessibility.Accessible)
              - RNAstructure\_java\_utilities.HTMLCheckBox
        - javax.swing.JPanel (implements javax.accessibility.Accessible)
          - RNAstructure\_java\_utilities.BorderedButtonPanel
          - RNAstructure\_java\_utilities.FieldPanel
            - RNAstructure\_java\_utilities.FieldPanel.FilePanel
            - RNAstructure\_java\_utilities.FieldPanel.NumberPanel
          - RNAstructure\_java\_utilities.RadioButtonPanel
        - javax.swing.JScrollPane (implements javax.accessibility.Accessible, javax.swing.ScrollPaneConstants)
          - RNAstructure\_java\_utilities.ScrollerPane
        - javax.swing.text.JTextComponent (implements javax.accessibility.Accessible, javax.swing.Scrollable)
          - javax.swing.JTextField (implements javax.swing.SwingConstants)
            - RNAstructure\_java\_utilities.FileField
            - RNAstructure\_java\_utilities.NumberField
              - RNAstructure\_java\_utilities.NumberField.DoubleField
              - RNAstructure\_java\_utilities.NumberField.FloatField
              - RNAstructure\_java\_utilities.NumberField.IntegerField
      - java.awt.Window (implements javax.accessibility.Accessible)
        - java.awt.Dialog
          - javax.swing.JDialog (implements javax.accessibility.Accessible, javax.swing.RootPaneContainer, javax.swing.WindowConstants)
            - RNAstructure\_java\_utilities.ValueSelectionDialog
  - RNAstructure\_java\_utilities.FileChooser (implements java.io.Serializable)
  - RNAstructure\_java\_utilities.ImageGrabber (implements java.io.Serializable)
  - RNAstructure\_java\_utilities.MacChecker (implements java.io.Serializable)
  - RNAstructure\_java\_utilities.SimpleDialogHandler (implements java.io.Serializable)

## Enum Hierarchy

- java.lang.Object
  - java.lang.Enum<E> (implements java.lang.Comparable<T>, java.io.Serializable)
    - RNAstructure\_java\_utilities.FileChooser.Types

- Overview
- Package
- Class
- Tree
- Index
- Help

- Prev
- Next

- Frames
- No Frames

- All Classes
